# Land use and land cover change effect on surface temperature over Eastern India

**DOI:** 10.1038/s41598-019-45213-z

**Published:** 2019-06-20

**Authors:** Partha Pratim Gogoi, V. Vinoj, D. Swain, G. Roberts, J. Dash, S. Tripathy

**Affiliations:** 1 School of Earth, Ocean and Climate Sciences, Indian Institute of Technology Bhubaneswar, Bhubaneswar, Odisha 752050 India; 2 Geography and Environmental Science, University of Southampton, Southampton, SO171BJ UK; 3 Department of Geology and Geophysics, Indian Institute of Technology Kharagpur, Kharagpur, India

**Keywords:** Atmospheric science, Climate change

## Abstract

Land use and land cover (LULC) change has been shown to have significant effect on climate through various pathways that modulate land surface temperature and rainfall. However, few studies have illustrated such a link over the Indian region using observations. Through a combination of ground, satellite remote sensing and reanalysis products, we investigate the recent changes to land surface temperature in the Eastern state of Odisha between 1981 and 2010 and assess its relation to LULC. Our analysis reveals that the mean temperature of the state has increased by ~0.3 °C during the past three decades with the most accelerated warming (~0.9 °C) occurring during the recent decade (2001 to 2010). Our study shows that 25 to 50% of this observed overall warming is associated with LULC. Further we observe that the spatial pattern of LULC changes matches well with the independently estimated warming associated with LULC suggesting a physical association between them. This study also reveals that the largest changes are linked to changing vegetation cover as evidenced by changes to both LULC classes and normalized difference vegetation index (NDVI). Our study shows that the state has undergone an LULC induced warming which accounts for a quarter of the overall temperature rise since 2001. With the expected expansion of urban landscape and concomitant increase in anthropogenic activities along with changing cropping patterns, LULC linked changes to surface temperature and hence regional climate feedback over this region necessitates additional investigations.

## Introduction

The surface temperatures are increasing globally as a consequence of anthropogenic climate change. However, it is known that observed changes are a result of both climate forcing and numerous other feedbacks including LULC. The LULC could change as a response to climate and also act as a feedback. In addition to these natural forcing and feedback cycles, there are also additional aspects that are linked to anthropogenic activities. This results in further modification to the LULC and meteorological responses thereupon^1–10^. These LULC changes and their effects are mostly discernible over regions having higher population density, industrialization, urbanization, deforestation, agricultural diversification etc. Thus, the most visible effect of anthropogenic activities regionally and locally are changes in the LULC which modifies the surface energy balance which in turn affects the surface temperature altering the region’s micro-climate^5,8,11–17^.

The changes in LULC also modulate the incidence of heat/cold waves, clouds and rainfall patterns^18–24^. In addition, LULC change have also been linked to atmospheric aerosol emissions^20,25,26^ which can modify the surface temperature through both direct and indirect effects, thereby modulating rainfall which can also result in droughts or floods through changes to extreme events under certain favorable circumstances^18^.

Over the Indian region, there are only a few scientific investigations that have attempted to discern LULC induced temperature changes, but they are either limited to the major metropolitan cities^6,11,20,23,27–33^ or have only focused on aspects related to urbanization^3,4,34–38^. For example, the surface temperature over western India is found to be warming by ~0.13 °C/decade due to the combined effect of greenhouse gases and LULC change of which ~50% was attributed to LULC change^27^. Also, in 2001 an area covering 26.4% of New Delhi had a diurnal temperature range (DTR) below 11 °C whereas in 2011 65.3% of New Delhi had a DTR below 11 °C which was attributed to the increase in built up area by 53%^24,29,31,39,40^. Furthermore, the LULC has also been linked to Indian monsoon rainfall changes^18,25^. Studies linking LULC to surface temperature changes are limited over Eastern India though this region is among the most rapidly changing landscape over the entire Indian region^41^. The region is also rich in mineral deposits and its continued exploitation for mineral wealth has accelerated LULC change in the past few decades. In addition, Odisha being one of the most natural disaster prone regions of India, a very few studies have investigated the relationship between LULC change and surface temperature, heat waves, extreme rainfall etc.

In this paper, we investigate the surface temperature changes over the state of Odisha using long term ground, satellite and reanalysis datasets and explore its relation to LULC changes. We investigate whether the surface temperature has increased and, if so, whether this is in response to changes in land cover and/or changes in climate. Then multiple line of evidences are used to link LULC to observed spatial and temporal pattern of temperature. This would help in establishing changes associated with local activities such as LULC and those due to regional and global climate change.

## Results and Discussion

### Trends in surface temperature

**Figure 1:**
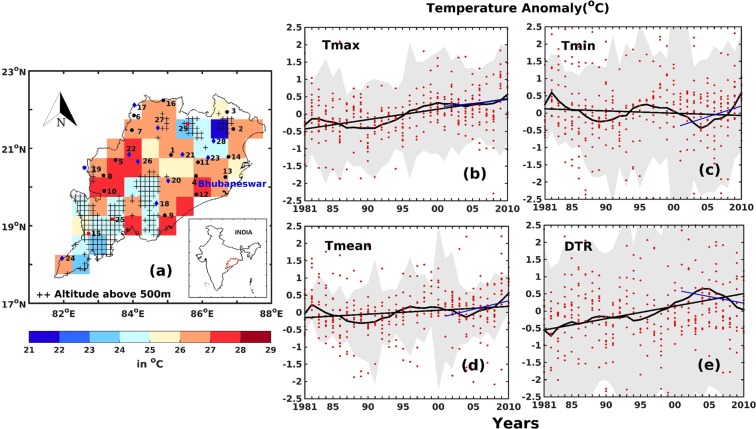
(**a**) The climatological annual mean surface temperature (°C) over Odisha during the period 1981 to 2010 (Source: University of Delaware). The inset map of the study area shows Odisha state with locations of all IMD observation sites (black) along with district headquarters (blue). The striped region represents areas that lie 500 m above mean sea level (Source: NGDC, NOAA). The numbers on the spatial plot represent serial numbers of the stations. (details are given in Supplementary Table S3) (**b**–**e**) shows the time series (10 point running mean) of the maximum, minimum, mean temperature and diurnal temperature range anomaly for all stations (averaged) within the plains (Source: IMD station datasets. See Table S3). The grey shaded region in (**b**–**e**) represent standard deviation. The map was generated using MATLAB 2015b, www.mathworks.com.

**Figure 2:**
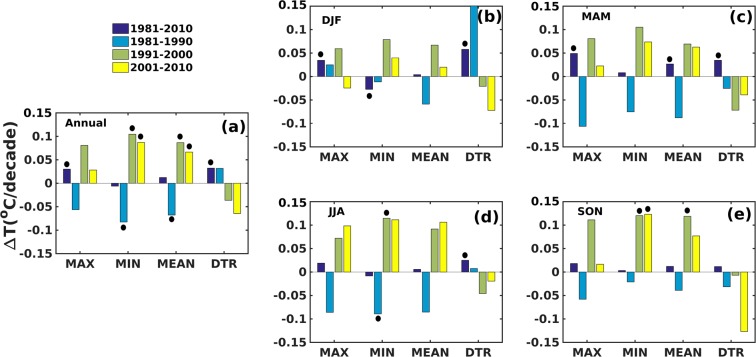
Decadal temperature trends over Odisha (**a**) Annual (**b**) December-January-February (DJF) (**c**) March-April-May (MAM) (**d**) June-July-August (JJA) (**e**) September-October-November (SON) (Source: IMD station datasets below 500 m amsl). Black dots in the plot represent statistical significance at 95% confidence level. The map was generated using MATLAB 2015b, www.mathworks.com.

The Indian subcontinent is characterized by large spatio-temporal variability in meteorological parameters displaying large annual, inter-annual, seasonal and decadal variability in surface temperature. In this section, the observed trends in temperatures both on an annual and inter-decadal basis are discussed. It is found that the state of Odisha had undergone a warming of ~0.3 °C during 1981 to 2010 and that the trend in surface temperature is positive irrespective of the meteorological station location (coastal or non-coastal) and altitude (high altitude or the plains). These trends were found to be statistically significant at 95% confidence level in most of the cases (Fig. 1 and Supplementary Information, Table S1). The temperatures were also found to have large inter-decadal variability. A separation into three decades (starting 1981, 1991 and 2001) shows that during the first decade (1981 to 1990) the mean temperature for sites below 500 m above mean sea level (amsl) decreased by ~−0.7 °C whereas, in the subsequent decades (1991 to 2000 and 2001 to 2010) an increase in temperature of ~1 °C and ~0.8 °C respectively was found (Fig. 2 and Table S1). Low values in the mean temperature trend are partly a consequence of differences in the maximum and minimum temperatures. For example, though maximum temperature was steadily increasing over the region, minimum temperature was found to be decreasing during 1981 to 1990. However, within these sub-periods, both of the recent decades show that minimum temperatures are increasing at different rates. It is evident that the recent two decades show a clear increase in both minimum (~1.2 °C) and maximum (~0.13 °C) temperatures which is reflected by the increasing mean temperature. However, the most interesting aspect is that the diurnal temperature range (DTR), which is observed to be increasing during the 1980’s by ~1.16 °C has decreased by ~−0.46 °C and ~−1.19 °C between the second (1991–2000) and third (2001–2010) decades respectively in areas below 500 m amsl (Table S1, Fig. 1b–e). These indicate that the overall trends are larger for low altitude stations in comparison to those at higher altitudes. In order to determine whether these findings are spatially consistent across the entire state or result from urbanization/LULC signatures we have used gridded climate datasets from IMD, University of Delaware (UDel) and NCEP/NCAR Reanalysis–1. Also, to assess the impact of El-Nino/Southern Oscillation (ENSO) events, years with extreme ENSO events (1982, 1983, 1997 and 1998) were removed from the analysis. It may be mentioned that the results with and without ENSO years are consistent (Supplementary Information, Table S5). Overall, our analysis reveals that the surface temperatures over the state has been increasing since 1981 (Fig. 1b–e).

### Seasonality of trends in surface temperature

We find that the observations and inferences made based on annual and decadal timescales are also applicable to seasonal timescales and indicate that the changes in temperature are forced on large spatial and temporal scales. Figure 2 indicates that between 1981 and 1990 the temperature reduced by 0.3 to 0.9 °C depending on season and this is evident both on annual and seasonal scales.

However, the region has warmed up since 1991 (~0.4 to 0.9 °C). It may be noted that analysis based on both gridded and station datasets show similar trends on cooling and warming during the first and subsequent two decades respectively. However the magnitude of the trends differ. The largest rate of increase in temperature is observed in the June to August (JJA) and September to November (SON) periods whilst the least is observed for December to February (DJF) period. Similar characteristics are also evident in the trends in maximum and minimum temperatures and that, irrespective of season, the minimum temperature is increasing at a much faster rate than the maximum temperature since 1991 (Fig. 2). Studies have shown that the rapid rate of change in minimum temperature over more than 70% of the global land surface could be linked to climate change^40^. However, those changes arising out of LULC are expected to be more localized in space and we therefore explore these temperature changes and their spatial patterns to assess the potential influence of land use.

### Spatial pattern of trends

**Figure 3:**
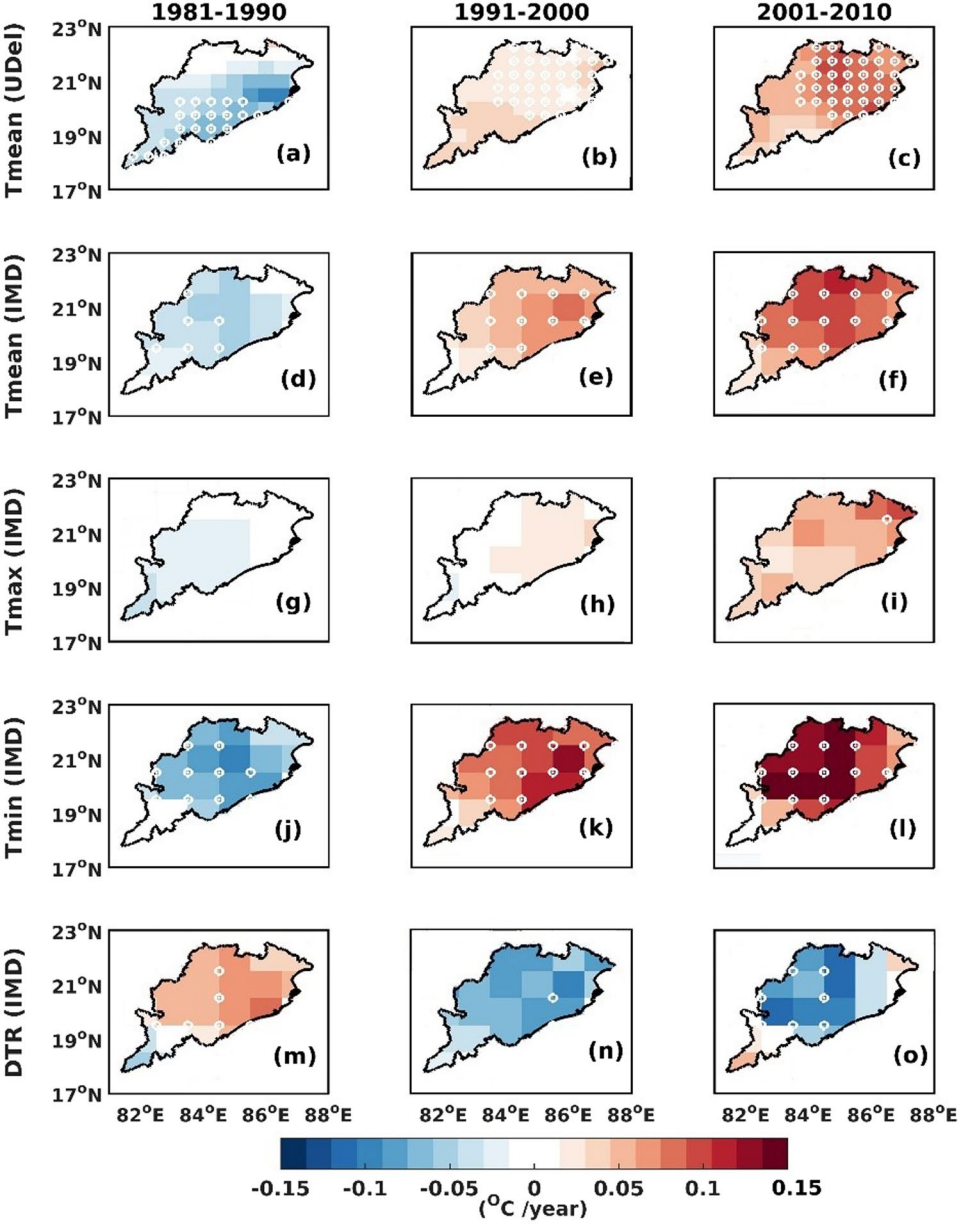
Decade-wise temperature trend of: (**a**–**c**)- Mean (University of Delaware) (**d**–**f**)- Mean (IMD) (**g**–i)- Maximum (IMD) (**j**–**l**)- Minimum (IMD) (**m**–**o**)- DTR (IMD). White circles in the plot represent statistical significance at 95% confidence level. The map was generated using MATLAB 2015b, www.mathworks.com.

Using the IMD gridded datasets, we find that the spatial pattern of the trends adhere to the inferences made using meteorological station datasets (Fig. 3).

Overall, the major finding is that between 1981 and 1990 a cooling trend is evident whereas the subsequent decades show the inverse to this. The spatial patterns and their temporal variability within different decades are similar for the average mean, minimum and maximum temperatures. In contrast, the diurnal temperature range (DTR) shows opposing trends with an increase during the decade starting 1981 and a decrease in the subsequent decades. In addition, the increase in minimum temperature is also more widespread spatially. This may indicate that the changes to surface temperature could be driven by climate change, overwhelming the LULC impacts. However, a warming trend is observed in the most recent decade over Odisha state and to determine whether this is linked to LULC change we utilize the widely used OMR technique which is detailed in the methodology section.

### The role of LULC in the observed warming

**Figure 4:**
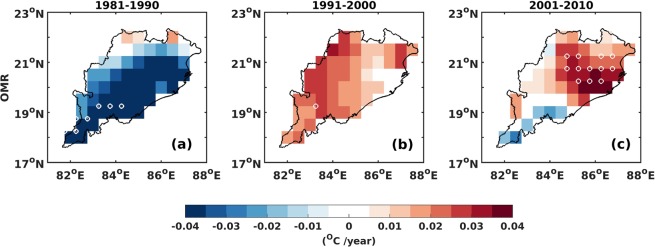
OMR trends over Odisha during the period (**a**) 1981–1990 (**b**) 1991–2000 (**c**) 2001–2010. White circles in the plot represent statistical significance at 95% confidence level. The map was generated using MATLAB 2015b, www.mathworks.com.

The OMR was calculated at a spatial resolution of 0.5° for the whole state of Odisha which is shown in Fig. 4 for the different decades. The first decade since 1981 displays a declining trend in OMR of ~−0.04 °C/year over the whole state. Though positive OMR are related to either urbanization or LULC linked changes, negative values are not directly related to these changes and could result from issues associated with the development of the reanalysis datasets that assimilate observations from surface and satellite measurements. The advent of satellite measurements and its assimilation in reanalysis or changes to the instrumentation, or both these factors combined could alter reanalysis results thereby impacting the OMR calculations and hence the negative values^42^. We therefore do not explore this decade further which is cooling since 1981 in our analysis. However, the past two decades since 1991 show a clear increasing trend of OMR over Odisha especially over West which shifts to the East during the most recent decade (since 2001). An interesting aspect is that the highest increasing trend of OMR (~0.04 °C/year) coincides with the location of cities such as Bhubaneswar and Cuttack (which are the densely populated cities) and are also to the East of the state (Figs 1a and 4c). The city of Bhubaneswar is among the fastest growing tier 2 cities in India and suggests that the OMR trend indicates the impact of LULC/urbanization. The OMR has been shown to be a robust method to detect urbanization/LULC impacts on surface temperatures^10,43–45^. To further strengthen this finding, we explore whether the highest OMR trends are coincident with the largest LULC changes. It may be noted here that further analysis are mostly carried out for the last decade due to availability of validated LULC dataset over the Indian region. In addition, even other supplementary datasets are expected to be better from various sources due to the availability/assimilation of data of highest quality since year 2000 from earth observing system (EOS) satellites.

### The LULC change analysis

**Figure 5:**
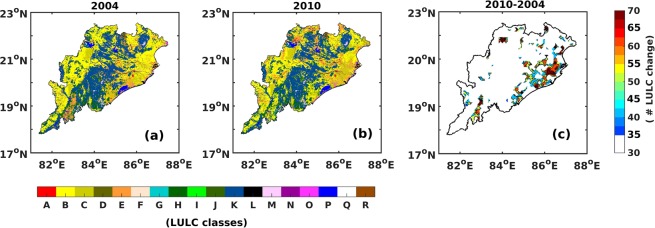
Land Use and Land Cover during (**a**) 2004 (**b**) 2010 (**c**) # LULC change (2010–2004) using AWiFS. The map was generated using MATLAB 2015b, www.mathworks.com. Classes: A-Urban/Built Up, B-Kharif Crop, C-Rabi Crop, D-Zaid Crop, E-Double Crop, F-Current Fallow, G-Plantation, H-Evergreen, I-Deciduous, J-Shurbland, K-Swamp, L-Grassland, M-Other Wasteland, N-Gullies, O-Scrubland, P-Water Bodies, Q-Snow, R-Shifting Cultivation. (Source: AWiFS LULC product of 1 km spatial resolution).

**Figure 6:**
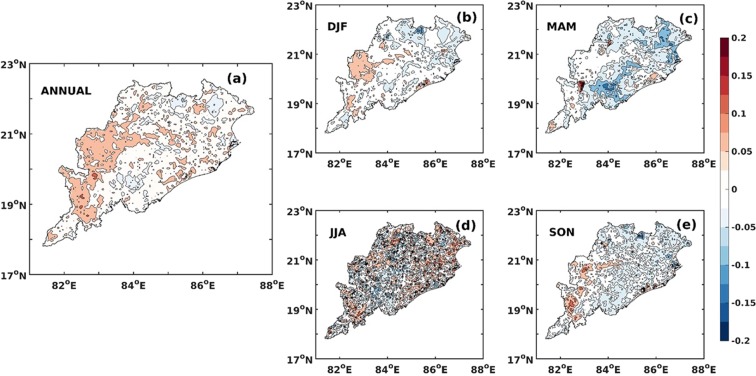
Trend of Normal Difference Vegetation Index (NDVI) during 2001 to 2010 (**a**) Annual (**b**) Winter (Dec to February, DJF) (**c**) Pre-monsoon (March to May, MAM) (**d**) Monsoon (June to August, JJA) and (**e**) Post-monsoon (September to November, SON). Source: MODIS –Terra (5.6 × 5.6 km). The map was generated using MATLAB 2015b, www.mathworks.com.

Our analysis reveals that the largest LULC changes occur over the NE part of the state (Fig. 5c) which shows the number of pixels that have undergone a change from the earlier classification (Fig. 5c) at a spatial resolution of 10 km. This was necessary due to heterogeneous land use and land cover change in the region and also to highlight the spatial extent of these changes better. We have therefore not specifically targeted any land use/cover type, but only investigated the land cover change during the study period. Therefore, the change analysis refers to those areas (number of pixels) where land has undergone change over the period 2004 to 2010. Overall, we find that the LULC change map matches well with the OMR trends shown in Fig. 4c during the recent decade since 2001. This provides us an independent confirmation that the OMR and its spatial pattern is due to temperature changes associated with LULC change. Now, the question is what caused these LULC changes? We therefore quantified individual LULC classes and their changes. It is found that there is a decrease in green vegetation over the state of Odisha (Supplementary Information, Fig. S1). We also carried out a detailed analysis to understand changes of different land use categories using the Advanced Wide Field Sensor (AWiFS) datasets between the periods 2004 to 2010 which coincides with the latest decade discussed in previous sections. Our analysis reveals (Supplementary Information, Fig. S1, Table S2) that Rabi Crop (October to March) cultivation has increased (~97%) over the state of Odisha during 2004 to 2010. This has occurred at the cost of decrease in the Kharif Crops (July to October) showing agricultural diversification and changing cropping patterns^41,46–48^. The largest changes are associated with vegetation (Kharif, Rabi crops, Fallow lands, Grasslands, Plantations etc.). Table S2 details the individual changes (in terms of area & percentages) to each LULC class. Several studies have also revealed the role of agriculture in changing the vegetation pattern thereby altering the characteristics of local meteorological parameters^19,49,50^. Our analysis using the NDVI for the period (2001 to 2010) further reveals consistent patterns, with large decrease in vegetation (Fig. 6) especially over the eastern part of the state.

This further confirms that the change in surface temperature are mostly a consequence of LULC change which is also evidenced by our change analysis using satellite based land cover classification. It may be mentioned that the change in NDVI pattern also coincides with the OMR pattern and LULC change pattern. These multiple line of evidences support the notion that the LULC change is associated with the changes to green cover and is related to vegetation or cropping patterns.

### Quantification of LULC linked temperature changes and urbanization

**Figure 7:**
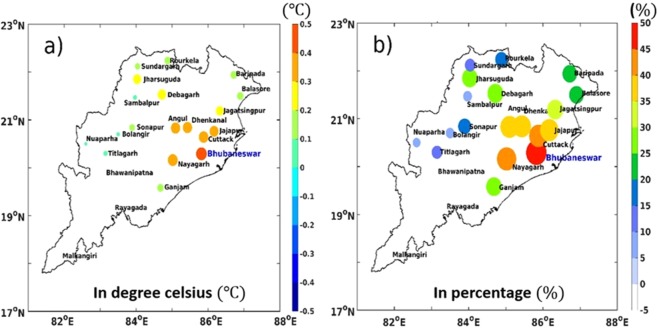
(**a**) Temperature rise during 2001–2010 due to LULC changes over Odisha (°C). (**b**) Percentage of temperature rise during 2001–2010 due to LULC changes over Odisha. (OMR in terms of percentage with respect to station observations). The map was generated using MATLAB 2015b, www.mathworks.com.

In the previous sections we found that the surface temperature increased due to land surface changes during the period 2001–2010 and is maximum over the eastern part of Odisha. The pattern of OMR, LULC and NDVI trends are all spatially coincident suggesting that the land use changes associated with green vegetation cover have led to the observed warming. However, urban growth may also alter temperature locally and to quantify relationship between LULC induced changes to temperature trends from the overall warming and to explore the signatures of urbanization, we calculated the percentages of the OMR’s LULC induced warming trends in relation to the total observed warming for all district headquarters over Odisha. The results indicate that the percentage of temperature rise due to OMR with respect to observations is highest over the urban centres. For example, Cuttack and Bhubaneswar being the most populous cities of Odisha experience temperature increase of ~40% and ~50% respectively during the period 2001–2010 (Fig. 7b) followed by Angul, Dhenkanal, Jajapur. The smaller rate of increase in the NNR dataset as compared to observation dataset in the past two decades has clearly signified that the surface temperature has increased mostly because of the LULC change. The largest rise for larger cities rather than smaller towns (Fig. 7a,b) highlights the additional impact of urbanization in the OMR analysis.

### Physical mechanism

The important parameters modulating LST are surface level soil moisture content and vegetation cover. Changes to these can alter the soil thermal properties and evapotranspiration. It is known that rise in the soil moisture leads to rise in the soil thermal capacity, conductivity and inertia thereby slowing the rise in the LST. In addition, surface heat fluxes such as the Latent Heat Flux (LHF) and Sensible Heat Flux (SHF) get modified with changes to land use. LHF (SHF) increases (decreases) with increasing vegetation leading to a decrease in LST^3–5,7,8,17,23,25,51–56^.

**Figure 8:**
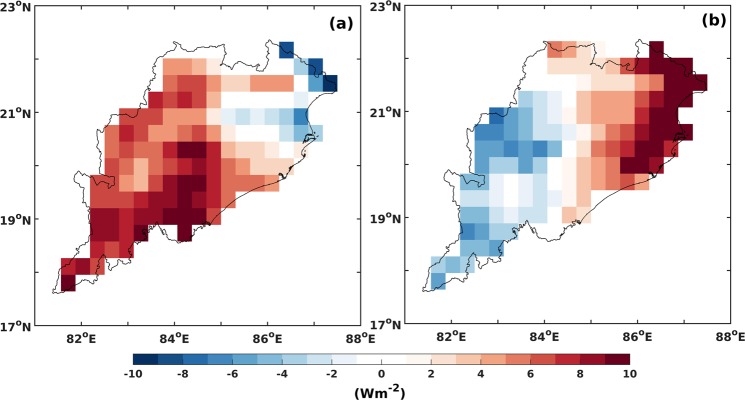
Change in (**a**) Latent Heat Flux and (**b**) Sensible Heat Flux during the period 2001–2010 (in Wm^−2^). Source: NCEP CFSv2 forecast product (30 km × 30 km). The map was generated using MATLAB 2015b, www.mathworks.com.

We therefore also explored changes to LHF and SHF. Our analysis reveals that the changes to both LHF (decreasing) and SHF (increasing) favors warming over Eastern part of Odisha (Fig. 8). Thus it can be confidently stated that the OMR patterns for the period 2001 to 2010, are consistent with those of the LULC, NDVI, SHF and LHF. Therefore, the spatial pattern of temperature changes during the most recent decade are primarily driven by LULC changes over the Eastern part of the state. However, the largest observed LULC linked changes are over the cities where urbanization further enhances the LULC signatures thereby showing the largest percentage wise increase in temperatures (Fig. 7).

## Summary and Conclusion

The LULC/urbanization induced surface temperature rise has become a common phenomenon all across the globe though the rate of change depends upon several external factors such as latitude, forest cover, soil type, mitigation practices etc. It may be noted that LULC change has been attributed to increased surface temperatures in Eastern China, USA, Europe, and India^24,27,28,31,36,39,43–45,57,58^. Despite varied locations, it is observed that the rate of increase in most of these places is ~0.1 °C/decade which is comparable to that of our study. Though the LULC induced warming has emerged over this region only in the past couple of decades, we find that in terms of LULC induced temperature rise, Eastern India is no less than any other developed regions in the world. This shows that more detailed investigations are urgently required to understand land use related changes to local and regional climate as several regions are undergoing rapid transformation as a result of developmental activities exacerbating the effects of modern climate change. Thus, for the first time over Eastern India this study has integrated surface, satellite and reanalysis datasets to reveal that,

1. The state of Odisha has warmed by about ~0.3 °C during the period 1981 to 2010 with accelerated warming of ~0.9 °C during the recent decade 2001 to 2010.
2. The minimum temperature is increasing at a rate higher than that of mean and maximum temperature since 2001 irrespective of location or altitude. It is also observed that there is a corresponding decrease in DTR during the recent decade.
3. A quarter of this warming is associated with LULC. However, over urban centers such as Bhubaneswar and Cuttack, this fraction is as high as half of the total warming.
4. There has been a general decreasing trend in NDVI over the Eastern part of the state during the period 2001 to 2010 associated with increasing SHF and decreasing LHF.
5. Overall the LULC induced warming is a result of changing vegetation cover. The changing cropping patterns (decreased Kharif and increased Rabi crops) appears to be the leading cause for these LULC changes which exacerbates the warming trend.

## Data and Methods

### Study domain

The state of Odisha with a population of ~42 million^59^ lies in the Eastern part of India, extending approximately from 81°E to 88°E and 17°N to 23°N (Fig. 1a) and is surrounded by the Bay of Bengal to the East and the Indian peninsula to the west. The region has a tropical climate resulting in high surface temperature during the months of April and May even leading to heat waves. On a climatological basis, most of the state has a mean temperature >26 °C annually with lower temperatures observed over high altitude locations (Fig. 1a). Odisha experiences an annual average rainfall of ~1500 mm primarily from the south-west monsoon during June to September^60^. In addition, it is also influenced by the monsoon depressions and tropical cyclones that makes landfall from the Bay of Bengal both during pre-monsoon and post-monsoon seasons.

### Datasets

A combination of station, satellite and reanalysis datasets over the past 30 years (1981 to 2010) are used to identify changes in surface temperature and its relationship to land use and land cover (LULC). The identification of LULC forced changes is based on the widely used observation minus reanalysis (OMR) technique. To characterize the change in temperature, we have used measurements of daily mean, maximum and minimum temperature at 29 stations (Fig. 1a and Table S3). In addition, we have also used daily gridded datasets from IMD^61,62^ and University of Delaware^63^ to explore the spatial patterns of temperature changes. The University of Delaware gridded mean temperature datasets were developed using surface measurements such as those from Global Historical Climate Network^64,65^ (GHCN). We have used NCEP-NCAR Reanalysis-1 (NNR) surface temperature datasets^66^ primarily for the calculation of OMR.

The land use classification and their change is inferred from the Indian Remote Sensing Satellite (IRS) satellite Resourcesat-1 (P6), Advanced Wide Field Sensor (AWiFS) derived gridded datasets (from ISRO’s Bhuvan data portal, https://bhuvan.nrsc.gov.in/) for the period 2004 and 2010. This is a gridded product developed and validated for use with mesoscale models^67^ for regional climate applications specifically over India. In addition, the topography dataset was obtained from National Geophysical Data Center (NGDC), NOAA to identify stations suitable for OMR analysis. This dataset was generated from the best available datasets which were further evaluated and edited before Digital Elevation Model (DEM) generation^68^.

The sensible and latent heat flux datasets from NCEP Climate Forecast System Version 2 (CFS v2) were used to detect changes in surface energy exchange. The NCEP CFS v2 is a consistent and stably calibrated forecast product. It provides a continuity of the climate data record with predictability of seasonal and sub-seasonal scale features^69^. The NDVI dataset were obtained from MODIS–Terra (MOD13C2) to determine changes in green vegetation cover. The MOD13C2 is derived using atmospherically corrected cloud free surface reflectance observations. Additional details about these datasets, their spatial and temporal resolution and period of observation are provided in Table S4 (Supplementary Information).

### Calculation of OMR and trends

To quantify the increase in temperature due to changes in LULC we have used Observation minus Reanalysis (OMR) technique developed by Kalnay and Cai (2003)^45^. This technique has been widely used to discern signatures related to land use changes and urbanization on surface temperature^10,17,27,43–45^ from observations. OMR relates the change in the temperature trend due to LULC by subtracting NCEP/NCAR Reanalysis-1 (NNR) from the observation. The NNR product^66^ was developed without assimilating surface parameters viz. surface temperature, moisture and winds^13,43–45^ effectively making it insensitive to local surface changes. Therefore, any trend in OMR may be attributed to the impact of urbanization or change in LULC^27,35,37,45,53,70,71^. The premise here is that observational trends are modulated by all processes including large scale (modern climate change) and local forcing such as LULC change, but the NNR product includes large scale forcing but not LULC change. Therefore, the difference in their trends will highlight the impact of LULC change.

The decadal trends in all parameters were calculated using a simple linear fitting tested against parametric student t-test for statistical significance. The observed changes/trends in station datasets are compared with both gridded and satellite retrieved products depending on availability and accessibility of the datasets during the study period for consistency.

## Supplementary information

Supplementary Material
