# Supplementary material for: Land use and land cover change effect on surface temperature over Eastern India
Source: Sci Rep. 2019 Jun 20;9:8859. doi: 10.1038/s41598-019-45213-z (PMC6586851; doi:10.1038/s41598-019-45213-z)
Supplement: Supplementary file 1 — Supplementary Material [file 41598_2019_45213_MOESM1_ESM.docx]

**Land use and land cover change effect on surface temperature over Eastern India**

Partha Pratim Gogoi^1^, V. Vinoj^1*^, D. Swain^1^, G. Roberts^2^, J. Dash^2^, S. Tripathy^3^

^1^School of Earth, Ocean and Climate Sciences, Indian Institute of Technology Bhubaneswar, Bhubaneswar, Odisha, INDIA – 752050.

^2^Geography and Environmental Science, University of Southampton, Southampton, UK SO171BJ.

^3^Department of Geology and Geophysics, Indian Institute of Technology Kharagpur, Kharagpur, INDIA

*Email Correspondence: [vinoj@iitbbs.ac.in](mailto:vinoj@iitbbs.ac.in)

**
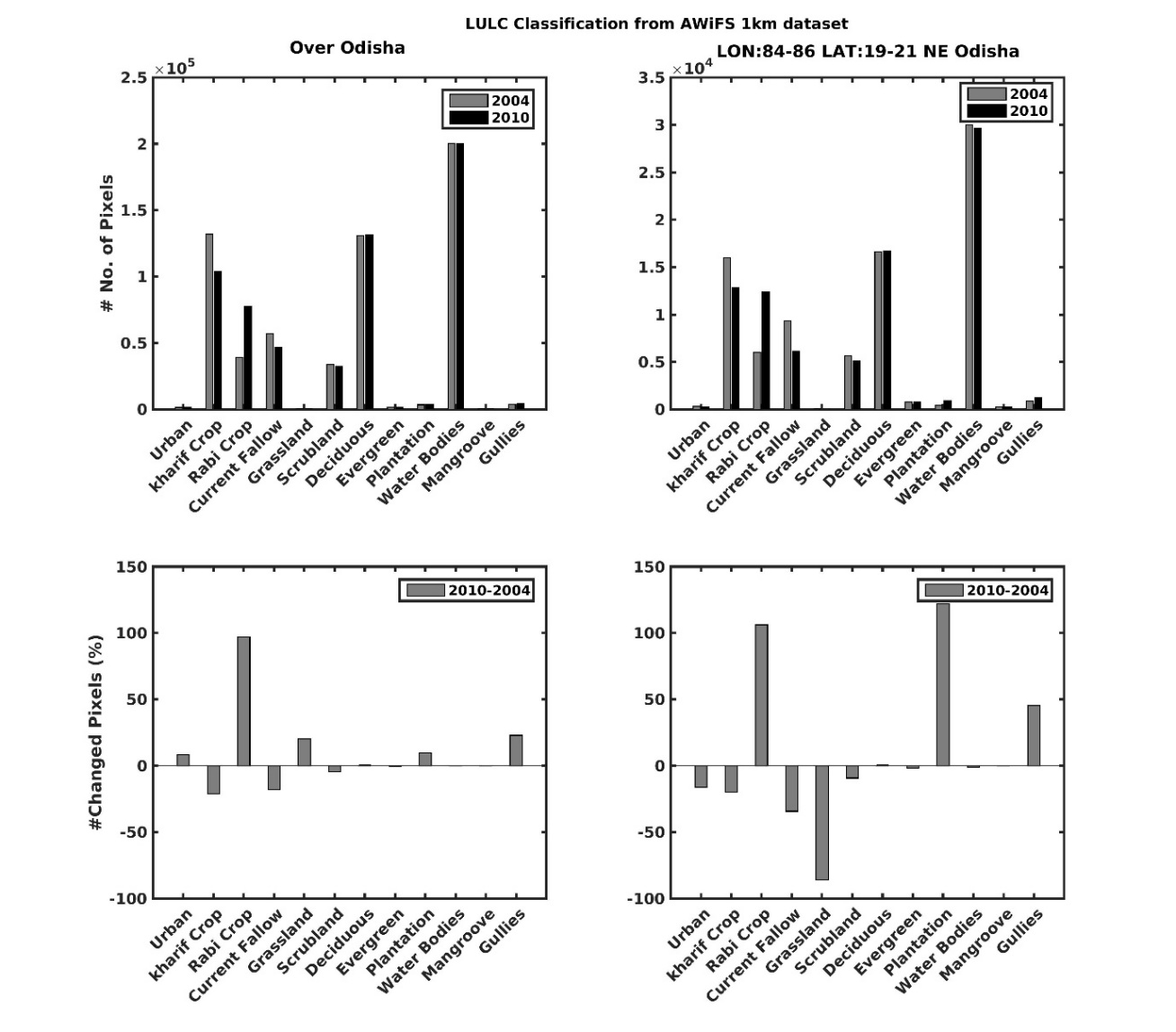
**

**Figure S1.** LULC classification and percentage change between 2004 and 2010. Source: AWiFS (1 $\times$1 km). The plots were generated using MATLAB 2015b, www.mathworks.com.

**
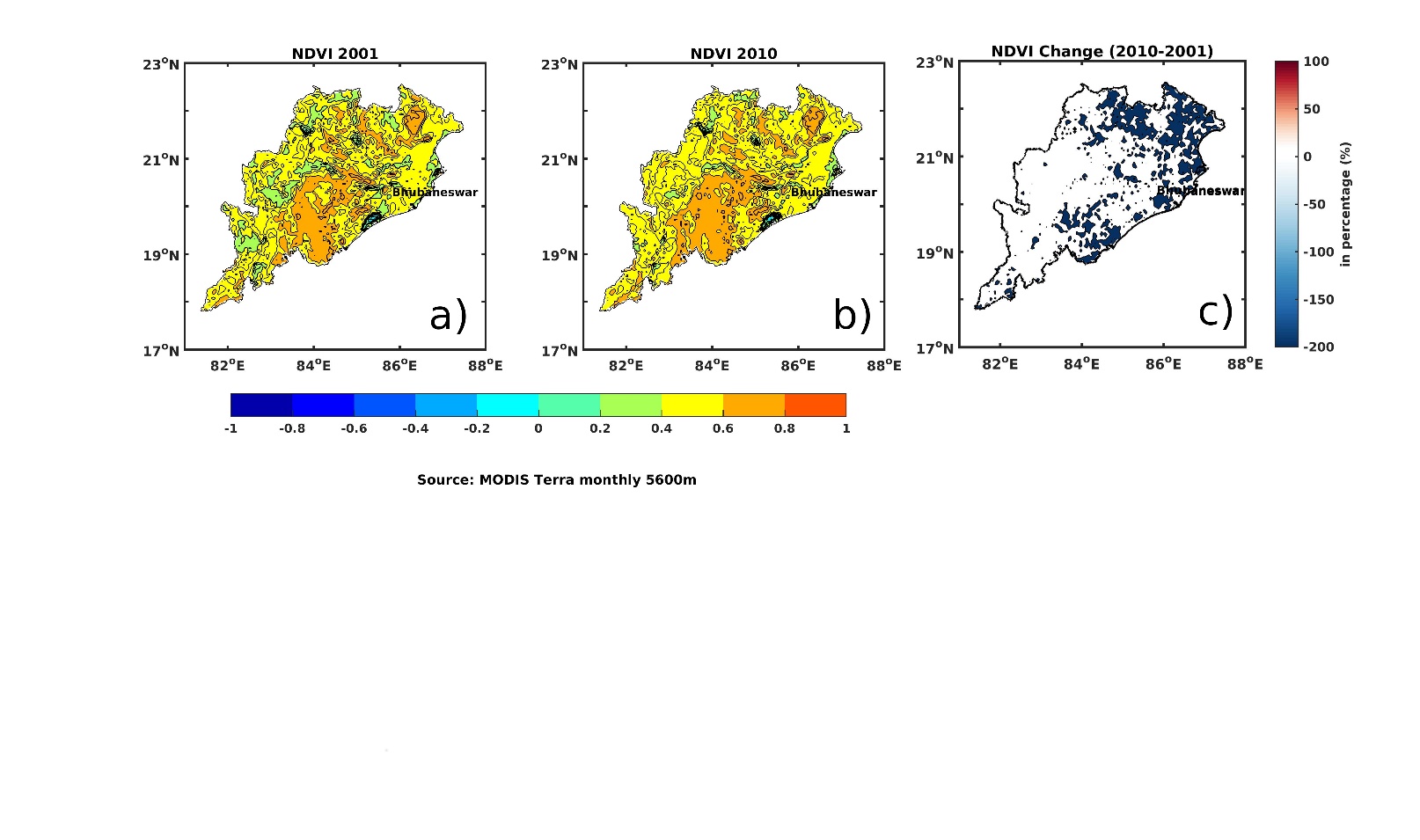
**

**Figure S2.** Annual Normal Difference Vegetation Index (NDVI) from (a) 2001 (b) 2010 (c) change in NDVI between 2001 and 2010 (in %) Source: MODIS Terra (5.6 $\times$ 5.6 km). The maps were generated using MATLAB 2015b, [www.mathworks.com](http://www.mathworks.com).


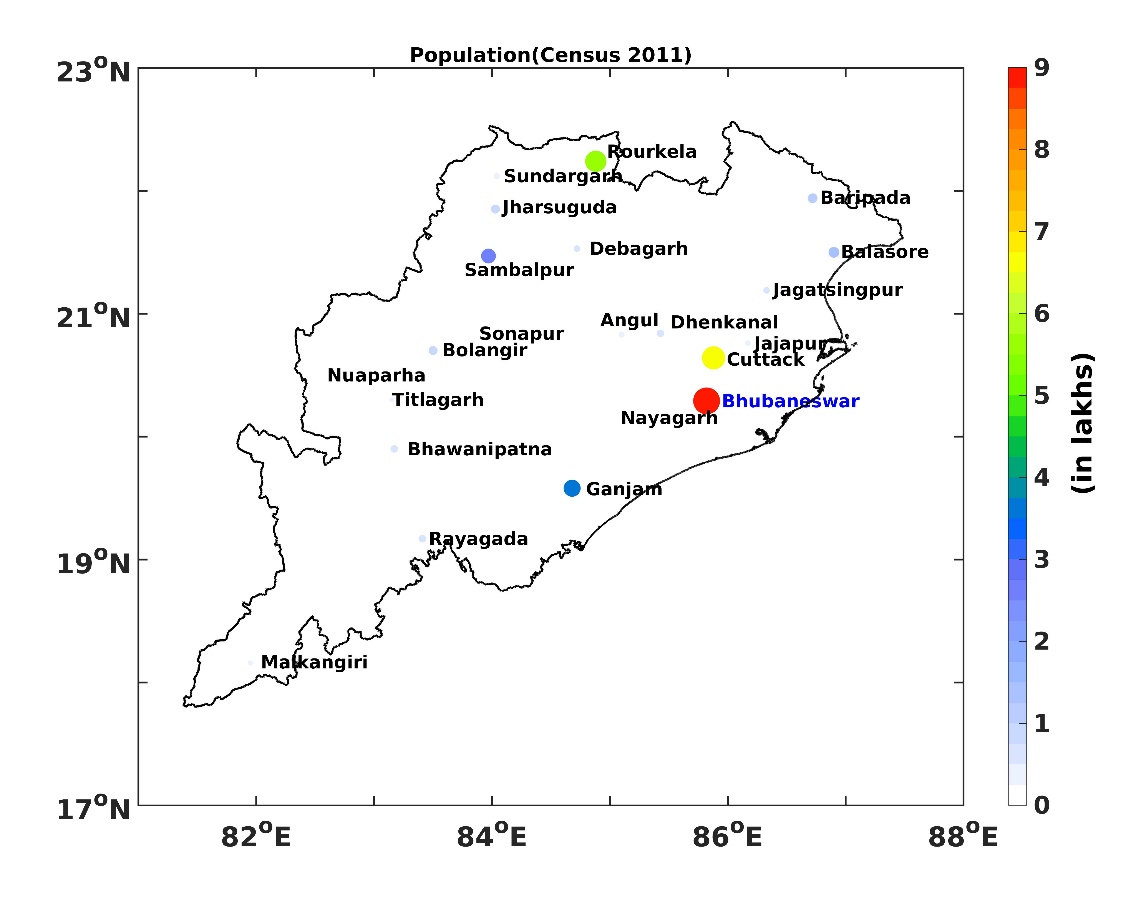


**Figure S3.** Station wise population (in lakhs, or 10^5^) over Odisha (Census 2011). The map was generated using MATLAB 2015b, [www.mathworks.com](http://www.mathworks.com).

**Table S1.**  Temperature change over Odisha (Source: IMD station datasets)

(Bold indicates statistical significance at 95% confidence level)

| **Sl. no.** | **Parameter** | **Data** | **1981-2010** | **1981-1990** | **1991-2000** | **2001-2010** |
| --- | --- | --- | --- | --- | --- | --- |
| **1** | **M**  **A**  **X** | **All Stations** | **0.70** | **-0.53** | **1.00** | **-0.07** |
|  |  | **Below 500 m** | **0.87** | **-0.56** | **1.17** | **0.13** |
|  |  | **No Coastal and Below 500 m** | **0.94** | **-0.81** | **1.53** | **0.10** |
| **2** | **M**  **I**  **N** | **All Stations** | **-0.53** | **-0.86** | **1.10** | **0.84** |
|  |  | **Below 500 m** | **-0.30** | **-0.88** | **1.29** | **1.24** |
|  |  | **No Coastal and Below 500 m** | **-0.91** | **-1.17** | **1.17** | **1.57** |
| **3** | **M**  **E**  **A**  **N** | **All Stations** | 0.08 | -0.68 | 0.84 | 0.47 |
|  |  | **Below 500 m** | **0.29** | **-0.70** | **1.01** | **0.81** |
|  |  | **No Coastal and Below 500 m** | 0.00 | -0.98 | 1.10 | 0.94 |
| **4** | **D**  **T**  **R** | **All Stations** | **1.25** | **0.28** | **-0.41** | **-0.10** |
|  |  | **Below 500 m** | **1.16** | **0.27** | **-0.46** | **-1.19** |
|  |  | **No Coastal and Below 500 m** | **1.90** | **0.25** | **-0.01** | **-1.55** |

**Table S2.** LULC classification and change in area (sq.km) and no. of pixels (%) between 2004 and 2010. (Data Source: AWiFS, 1 $\times$1 km Spatial Resolution)

| Sl. no. | LULC  Classes  (AWiFS) | Area Changed (km^2^) | | Change of pixels (%) | |
| --- | --- | --- | --- | --- | --- |
|  |  | Odisha  (2010-2004) | NE Odisha  (2010-2004) | Odisha  (2010-2004) | NE Odisha  (2010-2004) |
| 1 | Urban | 124 | -51 | 8.37 | -16.19 |
| 2 | Kharif Crop | -28043 | -3158 | -21.24 | -19.95 |
| 3 | Rabi Crop | 38109 | 6400 | 97.07 | 106.17 |
| 4 | Current Fallow | -10981 | -3210 | -18.02 | -34.29 |
| 5 | Grassland | 86 | -49 | 20.37 | -85.96 |
| 6 | Scrubland | -1456 | -522 | -4.29 | -9.20 |
| 7 | Deciduous | 541 | 72 | 0.41 | 0.43 |
| 8 | Evergreen | -7 | -15 | -0.43 | -1.96 |
| 9 | Plantation | 341 | 507 | 9.70 | 121.87 |
| 10 | Water Bodies | -267 | -362 | -0.13 | -1.20 |
| 11 | Mangroove | 0 | 0 | 0 | 0 |
| 12 | Gullies | 867 | 388 | 22.96 | 45.43 |

**Table S3.** Locations of all the stations in Odisha used in the study. IMD stations are shown in bracket.

| **Sl. No.** | **Stations** | **Latitude** | **Longitude** |
| --- | --- | --- | --- |
| 1 | Angul (IMD) | 20.83 | 85.1 |
| 2 | Balasore (IMD) | 21.5 | 86.9 |
| 3 | Baripada (IMD) | 21.94 | 86.72 |
| 4 | Bhubaneswar (IMD) | 20.29 | 85.82 |
| 5 | Bolangir (IMD) | 20.7 | 83.5 |
| 6 | Jharsuguda (IMD) | 21.85 | 84.03 |
| 7 | Sambalpur (IMD) | 21.47 | 83.97 |
| 8 | Titlagarh (IMD) | 20.3 | 83.15 |
| 9 | Gopalpur (IMD) | 19.27 | 84.92 |
| 10 | Bhawanipatna (IMD) | 19.9 | 83.17 |
| 11 | Cuttack (IMD) | 20.64 | 85.88 |
| 12 | Puri (IMD) | 19.81 | 85.83 |
| 13 | Paradipport (IMD) | 20.26 | 86.67 |
| 14 | Chandbali (IMD) | 20.78 | 86.76 |
| 15 | Koraput (IMD) | 18.8 | 82.72 |
| 16 | Rourkela | 22.24 | 84.88 |
| 17 | Sundargarh | 22.12 | 84.04 |
| 18 | Ganjam | 19.58 | 84.68 |
| 19 | Nuaparha | 20.5 | 82.6 |
| 20 | Nayagarh | 20.16 | 85.02 |
| 21 | Dhenkanal | 20.84 | 85.43 |
| 22 | Sonapur | 20.84 | 83.89 |
| 23 | Jajapur | 20.76 | 86.17 |
| 24 | Malkangiri | 18.16 | 81.95 |
| 25 | Rayagada | 19.17 | 83.41 |
| 26 | Baudh | 20.66 | 84.14 |
| 27 | Debagarh | 21.53 | 84.72 |
| 28 | Jagatsingpur | 21.19 | 86.33 |
| 29 | Keonjhargarh (IMD) | 21.63 | 85.58 |

**Table S4**. Details of all the datasets used in the study (type, spatial and temporal resolution, frequency of observation and source).

| **Sl. No.** | **Parameter** | **Type of data** | **Resolution** | | | **Source** |
| --- | --- | --- | --- | --- | --- | --- |
|  |  |  | **Spatial** | **Temporal** | **Period** |  |
| 1 | **Temperature (Max, Min and Mean)** | In-situ observation | Multiple Stations | Daily | 1981 to 2010 | India Meteorological Department |
| 2 | **Temperature (Max, Min, Mean)** | Gridded | 1$^{\circ}$ $\times$ 1$^{\circ}$ | Daily | 1981 to 2010 | India Meteorological Department |
| 3 | **Temperature (Mean)** | Gridded | 0.5$^{\circ}\times$ 0.5$^{\circ}$ | Monthly | 1981 to 2010 | University of Delaware |
| 4 | **Temperature (Mean)** | Gridded | 2.5$^{\circ}\times$ 2.5$^{\circ}$ | Monthly | 1981 to 2010 | NCEP-NCAR |
| 5 | **LULC** | Gridded | 1$\times$1 km | Yearly | 2004 & 2010 | RESOURCESAT-1(IRS-P6), ISRO |
| 6 | **Elevation/Topography** | Gridded | 1$\times$1 km | - | - | (NGDC, NOAA) |
| 7 | **Heat Flux (Latent, Sensible)** | Gridded | 30$\times$30 km | Monthly | 2001 to 2010 | NCEP CFSv2 |
| 8 | **NDVI** | Gridded | 5.6 $\times$ 5.6 km | Monthly | 2001 to 2010 | MODIS (Terra) |

**Table S5**. Temperature change over Odisha (Source: IMD station datasets). **I**- All years below 500 m & **II**- Strong ENSO years eliminated. (Bold indicates statistical significance at 95% confidence level).

| **Change (in** $\boldsymbol{℃}$**)** | **1981-2010** | | **1981-1990** | | **1991-2000** | | **2001-2010** | |
| --- | --- | --- | --- | --- | --- | --- | --- | --- |
|  | **I** | **II** | **I** | **II** | **I** | **II** | **I** | **II** |
| **T_mean_** | **+0.3** | **+0.57** | **-0.66** | **-0.1** | **+0.91** | **+1.08** | **+0.46** | **+0.46** |
| **T_max_** | **0.87** | **1.26** | **-0.54** | **-1.26** | **0.85** | **1.06** | **0.16** | **0.16** |
| **T_min_** | **-0.24** | **-0.09** | **-0.80** | **-0.74** | **1.10** | **1.25** | **0.64** | **0.64** |
| **T_max_- T_min_** | **1.0** | **1.26** | **0.31** | **-0.45** | **-0.36** | **-0.34** | **-0.47** | **-0.47** |
